# A B-Cell Superantigen Induces the Apoptosis of Murine and Human Malignant B Cells

**DOI:** 10.1371/journal.pone.0162456

**Published:** 2016-09-07

**Authors:** Daniela Lorenzo, Alejandra Duarte, Juliana Mundiñano, Paula Berguer, Irene Nepomnaschy, Isabel Piazzon

**Affiliations:** 1 IMEX-CONICET, Academia Nacional de Medicina de Buenos Aires, CABA, Argentina; 2 Fundación Instituto Leloir, IIBBA, CONICET, Buenos Aires, Argentina; University of Manitoba, CANADA

## Abstract

B-cell superantigens (Sags) bind to conserved sites of the VH or VL regions of immunoglobulin molecules outside their complementarity-determining regions causing the apoptosis of normal cognate B cells. No attempts to investigate whether B-cell Sags are able to induce the apoptosis of cognate malignant B cells were reported. In the present study we show that protein L (PpL), secreted by *Finegoldia magna*, a B-cell Sag which interacts with κ+ bearing cells, induces the apoptosis of murine and human κ+ lymphoma B cells both *in vitro* and *in vivo*. Apoptosis was not altered by caspase-8 inhibitor. No alterations in the levels of Bid, Fas and Fas-L were found suggesting that PpL does not activate the extrinsic pathway of apoptosis. The involvement of the intrinsic pathway was clearly indicated by: i) alterations in mitochondrial membrane potential (ΔΨm) both in murine and human lymphoma cells exposed to PpL; ii) decreased levels of apoptosis in the presence of caspase-9 inhibitor; iii) significant increases of Bim and Bax protein levels and downregulation of Bcl-2; iv) the translocation from the cytoplasm to the mitochondria of Bax and Bim pro-apoptotic proteins and its inhibition by caspase-9 inhibitor but not by caspase-8 inhibitor and v) the translocation of Bcl-2 protein from the mitochondria to the cytosol and its inhibition by caspase-9 inhibitor but not by caspase-8 inhibitor. The possibility of a therapeutic use of Sags in lymphoma/leukemia B cell malignancies is discussed.

## Introduction

Superantigens (Sags) are bacterial and viral proteins that have the capacity to affect immune responsiveness by interactions with highly represented conserved sites in the V regions of the antigen receptors of normal host lymphocytes, causing the apoptosis of cognate cells. T-cell Sags bind to major histocompatibility complex class II molecules as unprocessed proteins and subsequently interact with a high number of T cells expressing particular T cell receptor Vβ chains [1, 2]. B-cell Sags bind to conserved sites of the VH or VL regions of immunoglobulin (Ig) molecules outside their complementarity-determining regions causing the apoptosis of normal cognate B cells [3, 4]. Staphylococcal protein A (SpA) is the most studied B-cell Sag. SpA reacts with the Fabs of most V_H_3^+^ Igs, which are expressed on 30 to 60% of human peripheral B cells [3, 5].

Protein L (PpL), secreted by *Finegoldia magna* (previously termed *Peptostreptococcus magnus*) is a B-cell Sag that interacts with normal lymphocytes expressing the κ light chain in their B cell receptor (BCR). This interaction is restricted to products of Vκ1, Vκ3 and Vκ4 human genes [6]. It has been suggested that PpL is able to bind products of murine analogues of human Vκ 1, 3 and 4 families [6].

Other proteins defined as B-cell Sags include human immunodeficiency virus gp120, protein Fv (a human liver sialoprotein) and staphylococcal enterotoxin D [5, 7–9].

Superantigens [10, 11] and targeted T Sags [12, 13] have been used to enhance immunogenicity of murine and human tumor cells in different experimental models, mostly by fusing the Fab region of tumor-reactive monoclonal antibodies to mutated *Staphylococcus* enterotoxin A or *Staphylococcus* enterotoxin B. We have previously demonstrated that T-cell Sags are able to induce the apoptosis of cognate malignant T cells. We have shown that bacterial- and mouse mammary tumour virus (MMTV)-encoded Sags are able to induce the apoptosis of different murine-cognate lymphoma T cells both *in vitro* and *in vivo*, being the Fas-Fas-L pathway involved. *In vivo* exposure to bacterial T Sags significantly increased the survival of lymphoma-bearing mice. The permanent expression of a retroviral encoded-Sag induced the complete remission of an aggressive lymphoma in a high percentage of mice [14].

In our knowledge, no reports concerning the effects of B-cell Sags on B-cell malignancies have been reported.

In the present study we have investigated whether B-cell Sags are able to induce the apoptosis of cognate malignant B cells using spontaneous murine lymphoma B cells and human Daudi cells. We observed that PpL is able to induce the apoptosis of these malignant B cells being the mitochondrial pathway involved.

## Materials and Methods

### Mice

BALB/c mice were bred in the animal facility of the IMEX-CONICET, Academia Nacional de Medicina and all experimental procedures were carried out according to the policies of the Academia Nacional de Medicina, based on “Guide for Care and Use of Laboratory Animals. Bethesda, MD: National Institutes of Health; 1985”; NIH publication N 85–23. Experiments were approved by the ethical committee of the IMEX-CONICET (Permit number 1026).

### Spontaneous lymphomas and cell lines

LBK and LBO are spontaneous B-cell lymphomas that arose in old BALB/c mice from our laboratory [15]. Tumors were maintained by subcutaneous or intraperitoneal passages in syngeneic mice. Both tumours expressed CD19, CD5, IgM and low levels of IgD. LBK cells were κ+ and λ-; LBO was found to be κ- and λ+.

The mouse A20 cell line (TIB-208) was obtained from ATCC (Rockville, MD, USA). This line was established from a spontaneous reticulum cell neoplasm found in an old BALB/cAnN mouse and is κ+, λ-, CD19+ [16].

The human Daudi cell line (CCL-213) was obtained from ATCC (Rockville, MD). This cell line was established from a Burkitt´s lymphoma from a 16-year old boy. These cells were described to be EBV+, IgM+, κ+, λ- and CD19+ [17].

Daudi and A20 cells were maintained at 37°C in 5% CO_2_ in a humidified atmosphere in RPMI 1640 culture medium (GIBCO; Carlsbad, CA, USA) supplemented with 10% heat-inactivated FBS (GIBCO), 1% antibiotic-antimycotic (GIBCO) and 1% L-glutamine (GIBCO).

### Antibodies and dyes

For flow cytometry analysis (FACS) the following monoclonal antibodies (mAbs) and dyes were used: PE-coupled anti-human κ chain (clone 187.1; BD Pharmingen), FITC-coupled anti-human IgM (Clone R6-60.2; BD Pharmingen), FITC-coupled anti-mouse CD86 (clone B7-2; GL-1; BD Pharmingen), PE-coupled anti-mouse κ chain (clone G20-193; BD Pharmingen), FITC-coupled anti-mouse IgM (Clone II/41; BD Pharmingen), Annexin V (BD Pharmingen), propidium iodide (PI; Sigma-Aldrich; St. Louis, MO, USA), 3,3`- diethyloxacarbocyanine iodine (DiOC_2_(3)), 5,6 carboxifluorescein diacetate succinimidyl ester (CFSE; Molecular Probes; Eugene, OR, USA). For Western blot analysis the following antibodies were used: rabbit anti-human Bim, mouse anti-human Bax, rabbit anti-human Bcl-2, rabbit anti-human Bid (all from BD Pharmingen), mouse anti-human β-Actin (Cell signaling Technology; Danvers, MA, USA), For immunocytochemistry analysis the following secondary antibodies were used: goat Cy2-conjugated antibody directed against rabbit immunoglobulins and goat Cy3-conjugated antibody directed against mouse immunoglobulins (Invitrogen).

### Inhibition of caspase-3, -8 and -9

When indicated, Daudi cells were pretreated during 8hs with Caspase-9 Inhibitor III (Ac-LEHD-CMK), Caspase-8 Inhibitor II (Z-IE(OMe)TD(OMe)-FMK), Caspase-3 Inhibitor IV (Ac-DMQD-CHO; All Calbiochem) at 25μM final concentration in 1μl of DMSO. DMSO (1μl) was added in PBS and OVA controls.

### Flow cytometry

Cells (1×10^6^) were resuspended in RPMI 1640 without phenol red (GIBCO) containing 3% FBS, 0.1% sodium azide, and 10 mM HEPES (GIBCO), and incubated in one step with the appropriate mAbs [18]. Acquisition of 10.000–30.000 cells was performed using a FACScan or a FACSAria flow cytometer (BD Biosciences). Background values obtained with fluorochrome-conjugated isotype controls (BD Pharmingen) were subtracted. Results were analyzed using the CellQuest software (BD Immunocytometry Systems).

### Apoptosis assays

For *in vitro* assays, LBK, LBO, A20 or Daudi cells (1x10^5^) were cultured in 96-well flat-bottom plates and incubated in the presence of different doses of PpL, OVA or with PBS for 72 hs.

For PI staining, cells were washed, fixed with 1 ml of 70% ethanol and stored at 4°C for 24 hs. Cells were washed and resuspended in PBS containing 1% glucose, 1 mg/ml RNase A (Sigma), and 20 μg/ml PI. After 30 min of incubation, cells were acquired on a FACScan flow cytometer [18]. The percentage of cells with low DNA content was measured.

For *in vivo* assays, A20 cells were stained with 20 μM of CFSE [19] and 1x10^6^ cells were inoculated in the footpad of BALB/c mice. Twelve-twenty four hs later, animals (6 per group) were inoculated with 10 μg of PpL, 10 μg of OVA or PBS. After 12 hs the draining popliteal lymph nodes were excised and cells were stained with Annexin V. For this purpose, cells were washed with PBS, resuspended in 150 μl of Annexin V binding buffer and stained with 1 μl of Annexin V. After 30 min of incubation, cells were analyzed by FACS. The percentage of Annexin V+ cells within CFSE+ cells was determined.

### Mitochondrial membrane depolarization

To analyse changes in mitochondrial membrane potential (∆Ψm) [14] LBK, LBO, A20 and Daudi cells treated during 72 hs with PpL (60–100 μg/ml), OVA (60–100 μg/ml) or PBS were stained with DiOC_2_(3) at a final concentration of 10 nM in complete RPMI medium for 30 min at 37°C. Then, cells were washed twice with cold PBS and the percentage of DiOC_2_(3)^low^ cells was measured by FACS as indicative of mitochondrial depolarization.

### Protein isolation and Western blot

After different times of treatment with PpL, Daudi cells were collected and resuspended in RIPA buffer with protease inhibitors (Sigma-Aldrich). Total protein was measured by Bradford method. Extracts were separated on 15% SDS-PAGE and electrotransferred to Immun-Blot^TM^ PVDF Membrane (Bio-Rad). Membranes were incubated with blocking solution for 1 hour at room temperature with gentle shaking and incubated overnight with the primary antibody at appropriate dilutions: Anti-human Bim 1:1000; Anti-human Bax 1:1500; Anti-human Bcl-2 1:1000; Anti-human Bid 1:1500; Anti-human β-Actin 1:1000. After washed, membranes were incubated with HRP-conjugated secondary antibodies (Goat anti-Rabbit 1:5000, Goat anti-mouse 1:5000). The immunoreactive bands were detected with SuperSignal® West Pico Chemiluminescent Substrate (PIERCE, Thermo scientific; USA).

### Mitochondria isolation

Daudi cell were pretreated or not with caspase-9 or caspase-8 inhibitors. Eight hs latter, cells were treated with PpL (100 μg/ml), OVA (100 μg/ml) or PBS for 8hs. Cells were washed with PBS, resuspended in 10 mM Tris-HCl (pH 7.4), 250 mM sucrose, 0.1 mM EDTA, 10 μm leupeptin, 1 μm pepstatin A, and 1 mM EGTA (buffer A), homogenized and centrifuged at 600×*g* for 15 min. The supernatant obtained was centrifuged at 10000 ×*g* for 15 min and the mitochondrial pellet was washed once with buffer A and resuspended in 10 mM Tris-HCl (pH 7.4), 10 μm leupeptin (Sigma-Aldrich), 1 μm pepstatin A (Sigma-Aldrich), and 1 mM EGTA. The supernatant containing the cytoplasmic fraction and the mitochondrial fraction were used for protein measure.

### Total RNA isolation and RT-PCR

Total RNA was isolated using the RNA extraction Kit following the manufacturer´s instructions (Qiagen; Limburgo). RNA quantification was assessed with Gene Quant Pro (Amersham Biosciences; GE Healthcare, Buckinghamshire, UK). The reverse transcription and PCR analyses were carried out using Cloned AMV Reverse Transcriptase (Invitrogen; Carlsbad, CA, USA) according to the manufacture´s protocols. The generated cDNA were further amplified by PCR under optimized conditions using the primers pairs listed below with Recombinant Taq DNA Polymerase (Invitrogen). The primers used for isolation and amplification of the human Fas (hFas) were: sense primer 5`ACTGTATGTGAACACTGTGACCCT3`and antisense primer 5`CACTGTTTCAGGATTAAGGTTGG3`; for Bax: sense primer 5`CAGCTCTGAGCAGATCATGAAGACA3`and antisense primer 5`GCCCATCTTCTTCCAGATGGTTGAGC3`; for Bcl-2: sense primer 5´TTGAAGTGCCATTGGTAC3´ and antisense primer 5´CCAGCCTCCGTTATCCTG3´. For the comparison of the amount of amplified sequences produced from the different RNA samples, the amplified actin product of each sample was used as an internal standard, using the sense primer 5`TATGTGGGTGACGAGGCCCAGAG3`and antisense primer 5`TACTCCTGCTTGCTGATCCACATC3`. The reaction conditions were one cycle of 94°C for 40 sec, followed by 40 cycles of 94°C for 40 sec, 57°C for 60 sec and 72°C for 60 sec for Fas; 37 cycles of 94°C for 40 sec, 57°C for 60 sec and 72°C for 60 sec for Bax; 35 cycles of 94°C for 40 sec, 57°C for 60 sec and 72°C for 60 sec for Bcl-2 and 20 cycles of 94°C for 40 sec, 57°C for 60 sec and 72°C for 60 sec for actin. The number of cycles used was optimized for each gene to fall within the linear range of PCR amplification. PCR products were resolved on a 2% (wt/vol) agarose gel containing 0.5 μg/ml of ethidium bromide to determine the molecular sizes of the different amplicons. The gel images were acquired with the GelPro analyzer (IPS, North Reading, MA). The levels of the different mRNA amplicons were quantitated using a computer-assisted image analyzer (ImageQuant 5.2) and the PCR results for each sample were normalized by actin mRNA as an internal control.

### Immunofluorescence analysis

After 8 hs of incubation with PpL (100 μg/ml), OVA (100 μg/ml) or PBS Daudi cells were centrifuged by cytospin and fixed with 4% paraformaldehyde in PBS for 10 min at room temperature and permeabilized with 0.01% Triton X-100 for 10 min at 4°C. After several washes with 1% PBS-0.05% Tween-20, cells were blocked with 1% albumin in PBS-0.05% Tween-20 for 60 min at room temperature. Cells were incubated with the corresponding primary antibody overnight at 4°C (rabbit anti-human Bim polyclonal IgG (1∶500) and mouse anti-human complex III IgG (1:500)). After several washes, cells were incubated for 1 h at room temperature with the corresponding secondary Ab: goat Cy2-conjugated antibody directed against rabbit immunoglobulins (1:500) or goat Cy3-conjugated antibody directed against mouse immunoglobulins (1:500). Coverslips were mounted onto the slides using Fluorsave antifade reagent (Calbiochem, CA) followed by examination using a Zeiss LSM 510 laser-scanning confocal microscopy. Signal overlap was quantified using MBF-Image J and Pearsons colocalization coefficients were calculated.

### Statistical analysis

Levels of significance were determined using the Student’s *t* test or analysis of variance (ANOVA) followed by the Tukey post-test.

## Results

### PpL induces the activation of murine κ+ malignant B cells

**Fig 1:**
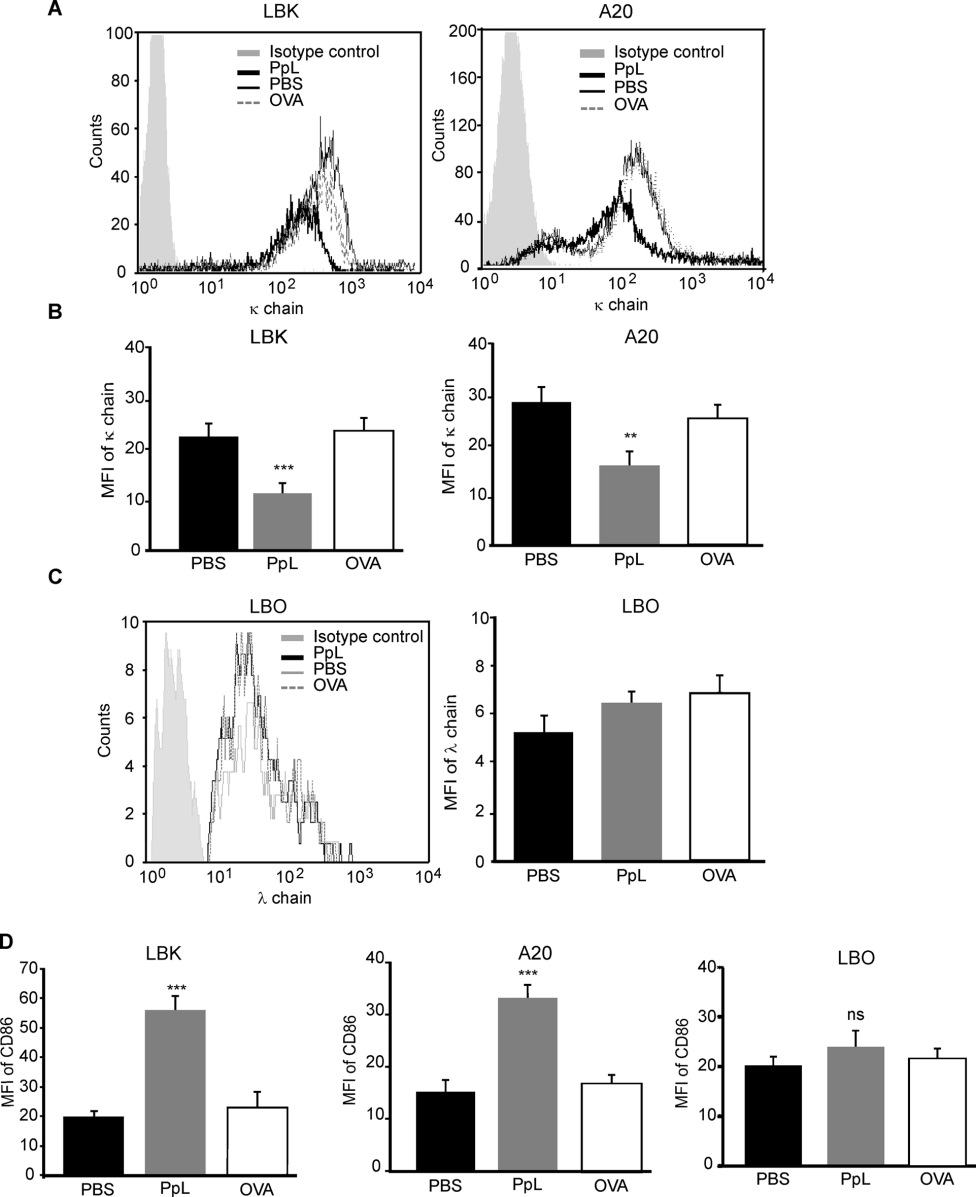
PpL decreases the expression of κ chain and of CD86 co-stimulatory molecule in κ+ murine lymphoma B cells. LBK, A20 and LBO cells were incubated in the presence of PpL (100 μg/ml), OVA (100 μg/ml) or PBS for 1 hr. The level of expression of the κ chain, λ chain or CD86 was measured by FACS. **A)** Representative overlaid histograms of κ chain expression in LBK and A20 cells. **B)** MFI of κ chain expression in LBK and A20 cells. **C)** Representative overlaid histograms and the MFI of λ chain expression in LBO cells. **D)** MFI of CD86 expression in LBK and A20 cells. Data are presented as the mean ± SEM of three independent experiments. *** p<0.001 PpL vs PBS, ** p<0.01 PpL vs PBS, ns p>0.05 PpL vs PBS).

In normal κ+ B cells, PpL decreases the level of expression of BCR, increases the levels of expression of co-stimulatory molecules [20–22] and induces apoptosis [6] (S1 Fig). In order to investigate whether PpL was able to induce alterations in the level of expression of the BCR receptor of murine B-cell lymphomas, A20, LBK, and the κ- LBO lymphoma cells were cultured in the presence of 100 μg/ml of PpL during 60 min. PpL was able to induce an early decrease in the mean fluorescence intensity (MFI) of the κ chain in these cells (Fig 1A and 1B). No alterations in the level of expression of the λ chain of the BCR were detected in LBO cells (Fig 1C). PpL did not induce the expression of the κ chain in LBO cells (S2 Fig). Furthermore, PpL was able to increase the levels of expression of co-stimulatory molecules such as CD86 (Fig 1D) and CD80 (data not shown) only in κ+ malignant murine B cells. These results suggest that the interaction between PpL and κ+ B-cell lymphomas is able to activate these cells as occurs with normal B cells.

### PpL increases the level of apoptosis of murine κ+ tumour cells both *in vitro* and *in vivo*

**Fig 2:**
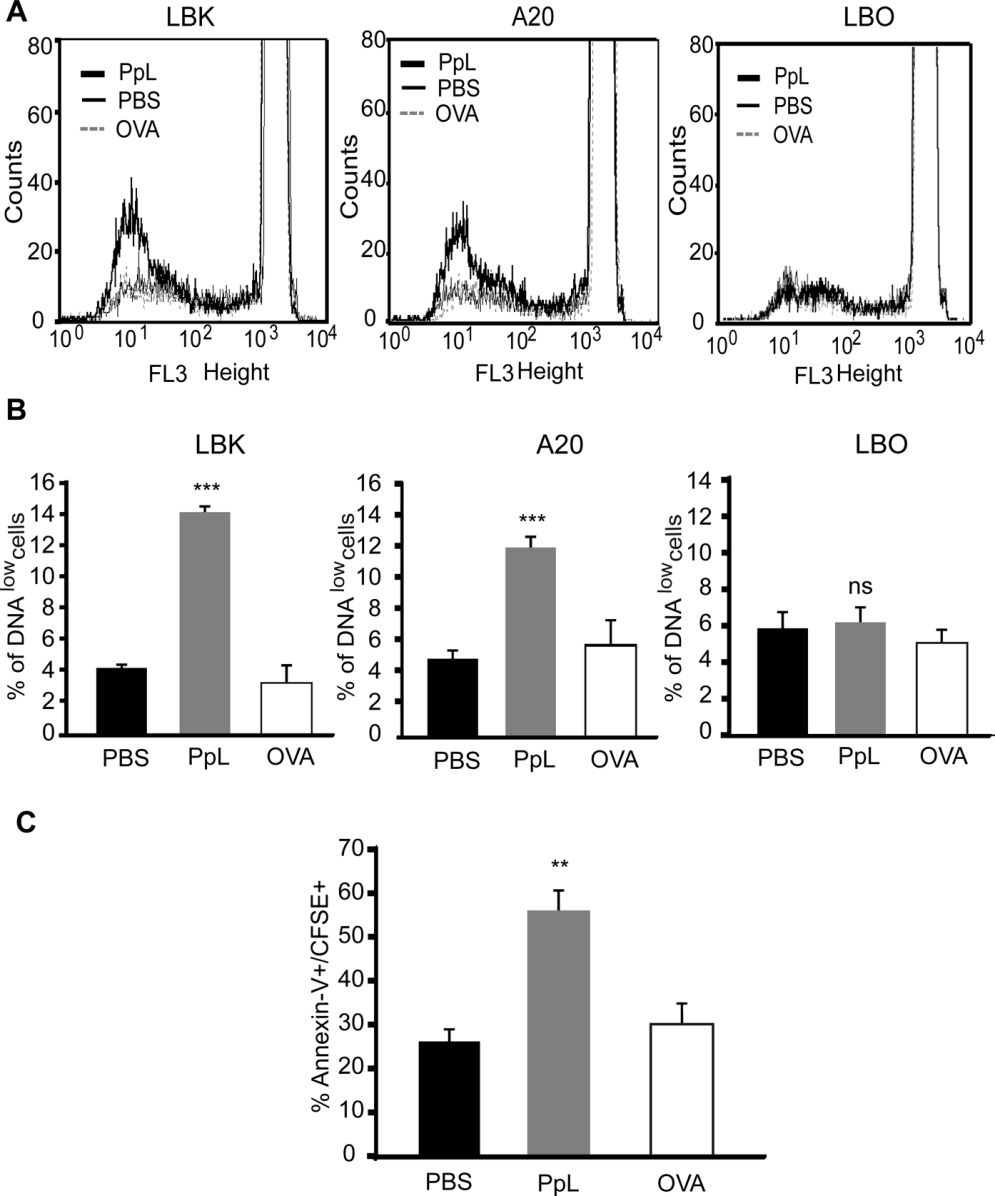
PpL induces the apoptosis of κ+ murine lymphoma B cells. **A-B) PpL decreases the DNA content of κ+ murine lymphoma B cells**. LBK, A20 and LBO cells were incubated in the presence of PpL (100 μg/ml), OVA (100 μg/ml) or PBS. After 72 hs, cells were labeled with PI and the DNA content was measured by FACS. **A)** Representative overlaid histograms of PI staining. **B)** Mean ±SEM of three independent experiments (*** p<0.001 PpL vs PBS.) **C)** *In vivo* PpL increases the apoptosis of A20 cells. A20 cells stained with CFSE were inoculated in the footpad of BALB/c mice. Twelve hs later animals were inoculated with PpL, OVA or PBS. After 12 hs popliteal lymph nodes were excised and cells were stained with Annexin V. The percentage of Annexin V+ within CFSE+ cells is shown. Data are presented as the mean ± SEM of three independent experiments, (** p<0.01 PpL vs PBS).

Murine lymphoma cells were stained with CFSE and cultured in the presence of PpL. No differences in the levels of proliferation were observed after 24–36 hs (data not shown). As already mentioned, PpL is able to induce the apoptosis of normal B cells expressing the κ chain [6]. *In vitro* assays were performed in order to determine whether PpL was able to induce a decrease in the DNA content of κ+ lymphoma B-cells. As can be observed in Fig 2A and 2B, the percentage of LBK and A20 cells with low DNA content increased in the presence of PpL. No alterations in the DNA content were registered in LBO cells (Fig 2A and 2B). These results suggest that PpL is able to induce the apoptosis of murine κ+ malignant B cells.

In order to investigate the *in vivo* effect of PpL on malignant cognate B cells, A20 cells stained with CFSE were inoculated in the footpad of BALB/c mice. Twelve to twenty four hs later, PpL was footpad-inoculated. Popliteal lymph nodes were excised 12 hs later and cells were stained with Annexin V. Results show that PpL increased the percentage of Annexin V+ cells within CFSE stained cells (Fig 2C), indicating that PpL is able to increase the level of apoptosis of malignant B cells *in vivo*.

**Fig 3:**
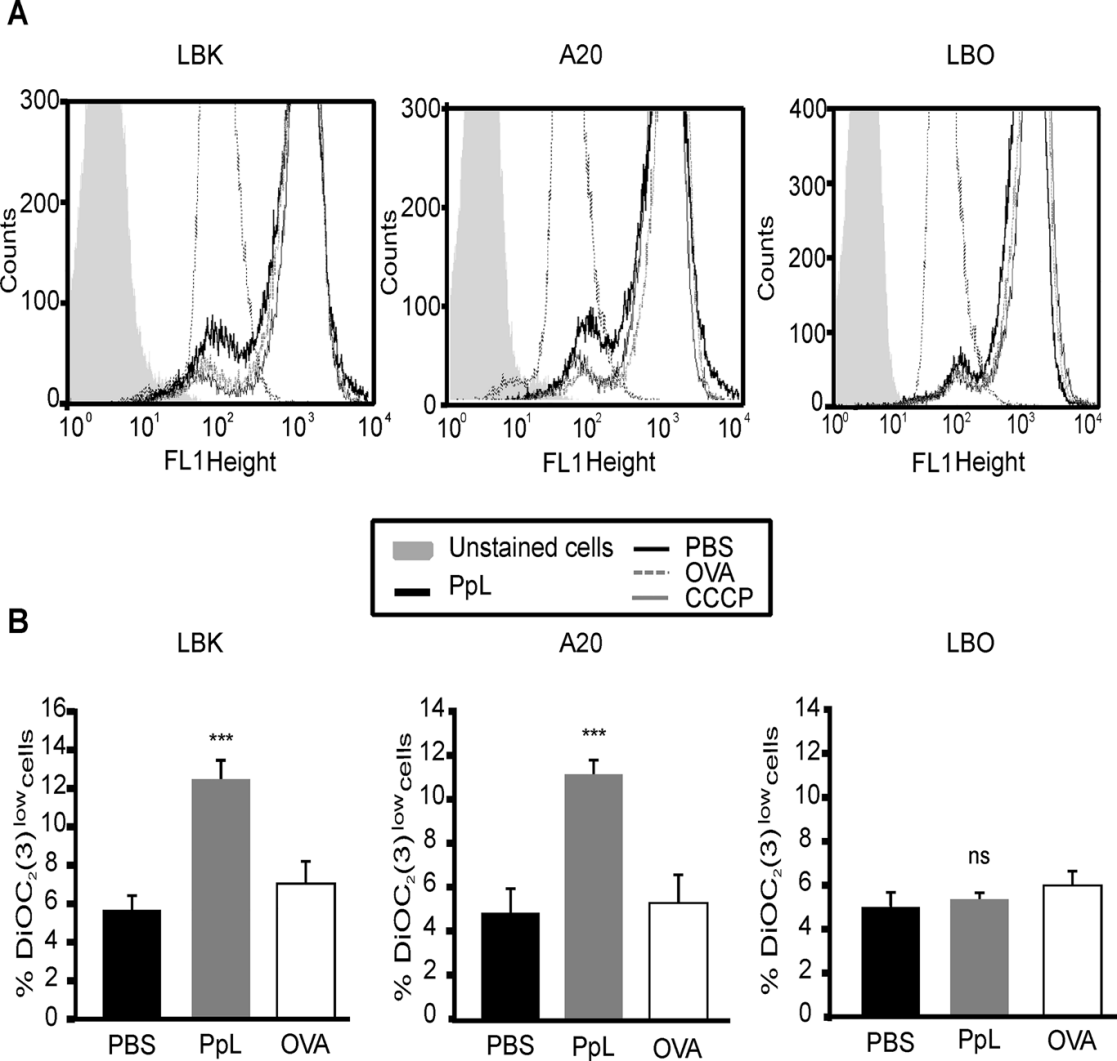
PpL causes mitochondrial depolarization in murine κ+ lymphoma B cells. LBK, A20 and LBO cells were incubated in the presence of PpL (100 μg/ml), OVA (100 μg/ml) or PBS. After 72 hs, the ΔΨm was measured by FACS using DiOC_2_(3) staining. **A)** Representative overlaid histograms for DiOC_2_(3) fluorescence. **B)** Percentage of DiOC_2_(3)^low^ cells. Data are expressed as mean ± SEM of three independent experiments, (*** p<0.001 PpL vs PBS; ns p>0.05 PpL vs PBS).

Because the intrinsic pathway is the route described for PpL-induced apoptosis in normal B cells [6, 23], we investigated if PpL was able to induce changes in the ΔΨm in murine lymphoma B cells. Results obtained show significant increases in the percentage of DiOC_2_(3)^low^ cells in LBK and A20 cells treated with PpL but not in LBO cells (Fig 3). These data suggest that the mitochondrial pathway is involved in the induction of apoptosis in malignant κ+ B cells by PpL.

### PpL induces the activation of Burkitt´s lymphoma cells

**Fig 4:**
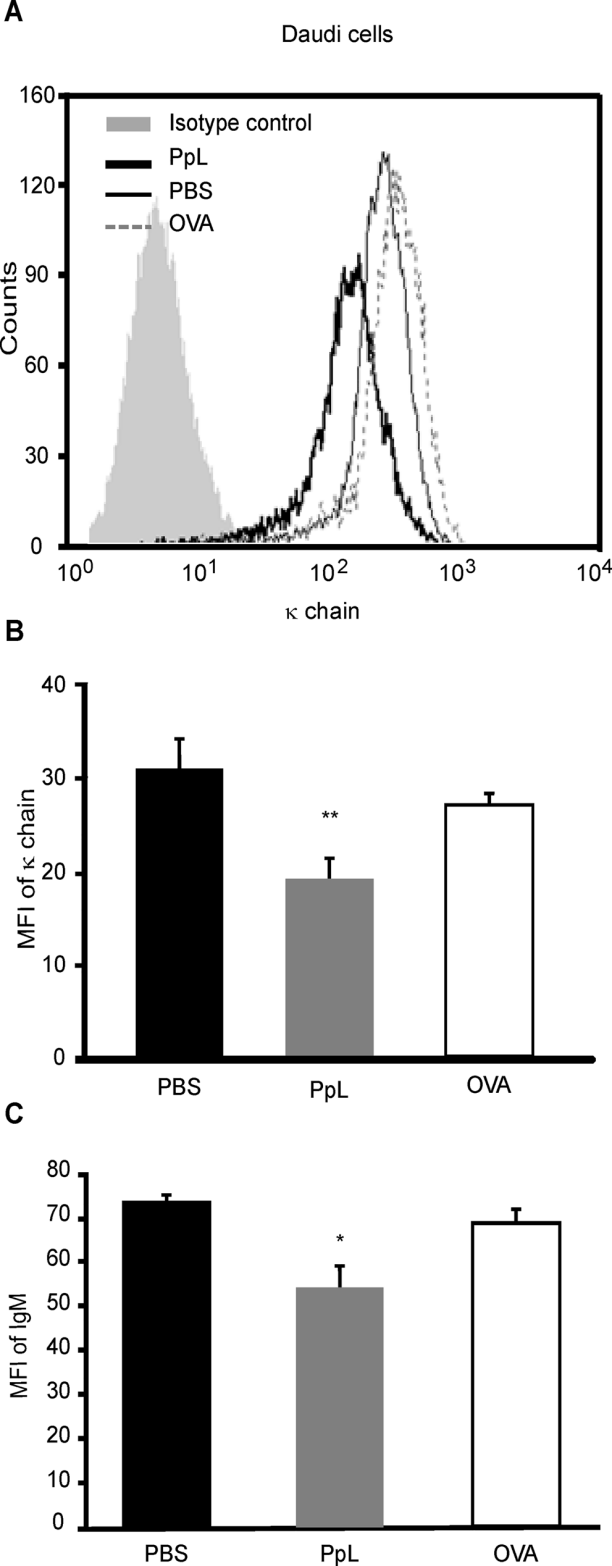
PpL decreases the expression of IgM and κ chain in a human malignant B cell line. Daudi cells were incubated with PpL (100 μg/ml), OVA (100 μg/ml) or PBS for 1hr and the expression of κ chain and IgM were measured by FACS. **A)** Representative overlaid histograms of κ chain expression. **B)** MFI of κ chain expression (mean ± SEM). **C)** MFI of IgM (mean ± SEM). The experiments were performed three times with similar results (* p<0.05 PpL vs PBS; * p<0.01 PpL vs PBS).

In order to investigate the *in vitro* effects of PpL in cognate (κ+) human malignant cells, Daudi cells were incubated with PpL and the levels of expression of IgM and κ chain were assessed. As can be observed in Fig 4, PpL was able to induce a decrease in the MFI of IgM and κ chain in these cells.

### PpL induces apoptosis in Burkitt´s lymphoma cells through the intrinsic pathway

**Fig 5:**
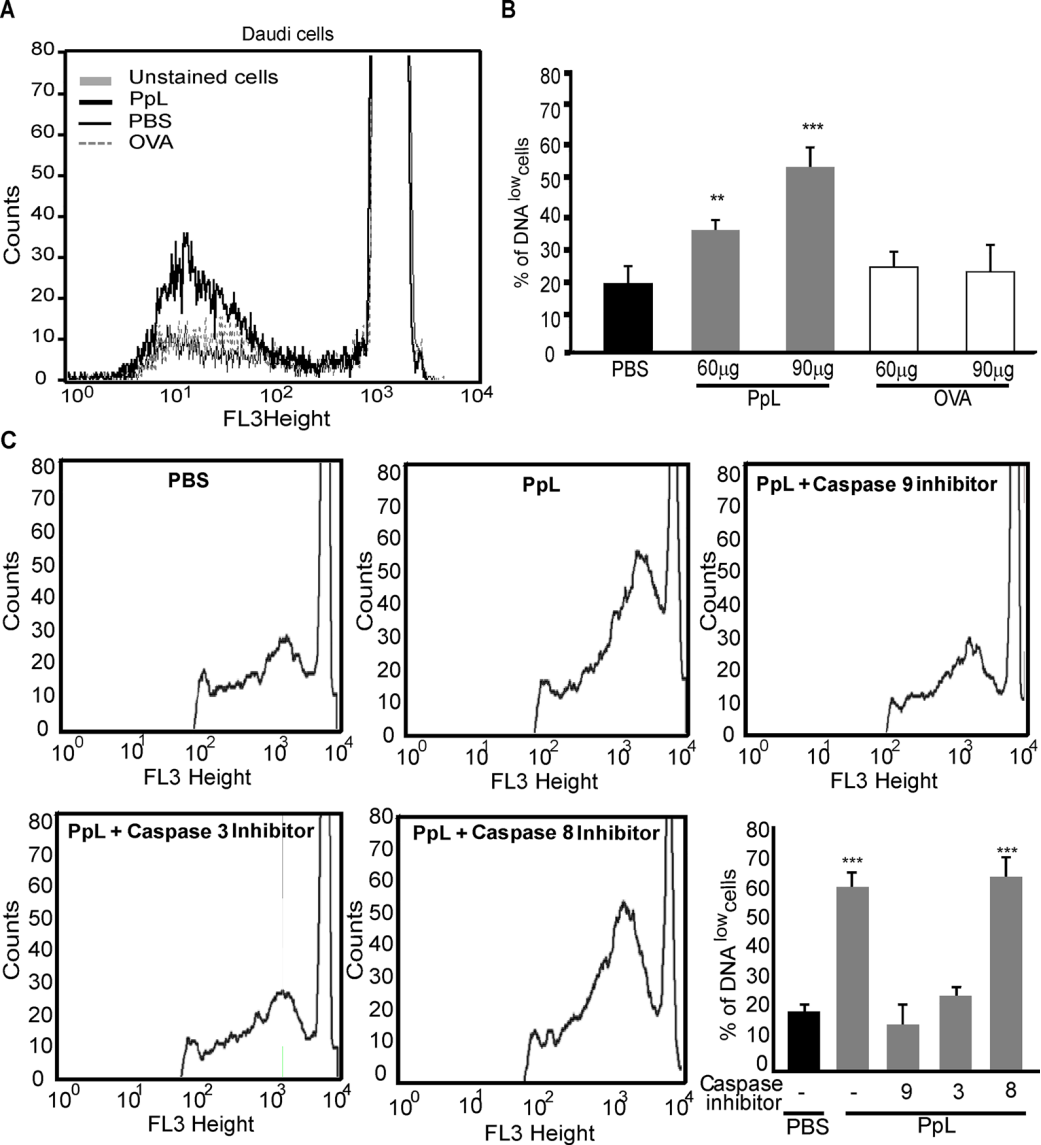
PpL decreases the DNA content and causes mitochondrial depolarization in Daudi cells being caspase-9 involved. **A-B)** Daudi cells incubated with different doses of PpL, OVA or PBS during 72 hs were labeled with PI. **A)** Representative overlaid histogram of the DNA content using 90 μg of PpL. **B)** Percentage of cells with decreased DNA content (mean ± SEM). Experiments were performed three times with similar results, (** p<0.01 PpL (60 μg/ml) vs PBS, *** p<0.001 PpL (90 μg/ml) vs PBS.). **C)** Representative histograms of the DNA content and the percentage of DNA low cells (mean ± SEM). Daudi cells pretreated or not with inhibitors of caspase-8, -9 or -3, were incubated with PpL (100 μg/ml), OVA (100 μg/ml), or PBS for 72 hs and labeled with PI. Experiments were performed three times with similar results, (*** p<0.001 PpL vs PBS and PpL+ caspase-8 inhibitor vs PBS).

**Fig 6:**
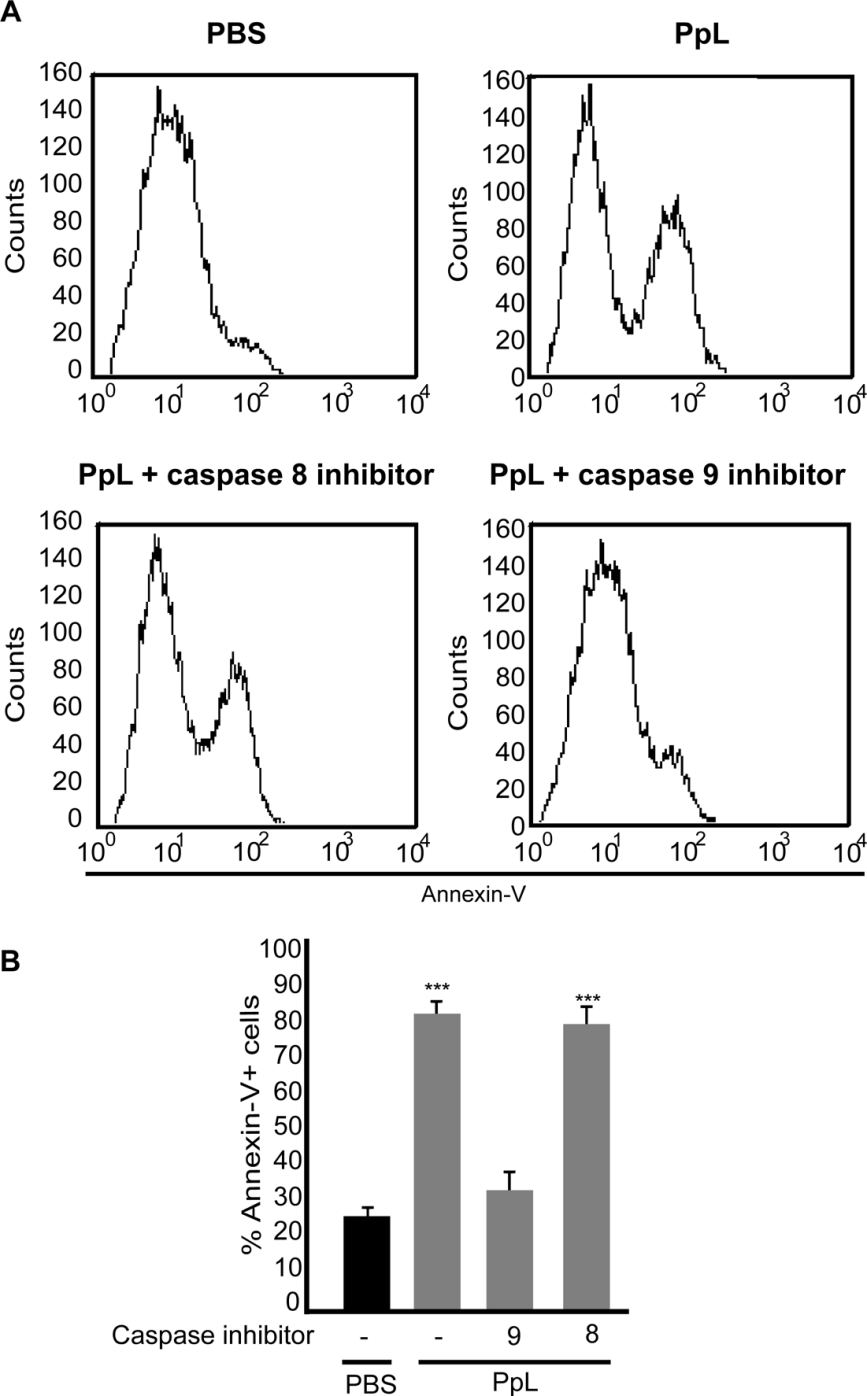
PpL increases the percentage of Annexin V+ Daudi cells. Daudi cells pretreated or not with caspase-8 or -9 inhibitors were incubated with 100 μg of PpL or PBS. After 72 hs, cells were stained with Annexin V. **A)** Representative histograms. **B)** Percentage of Annexin+ cells (mean ± SEM). Experiments were performed three times with similar results, (*** p<0.001).

**Fig 7:**
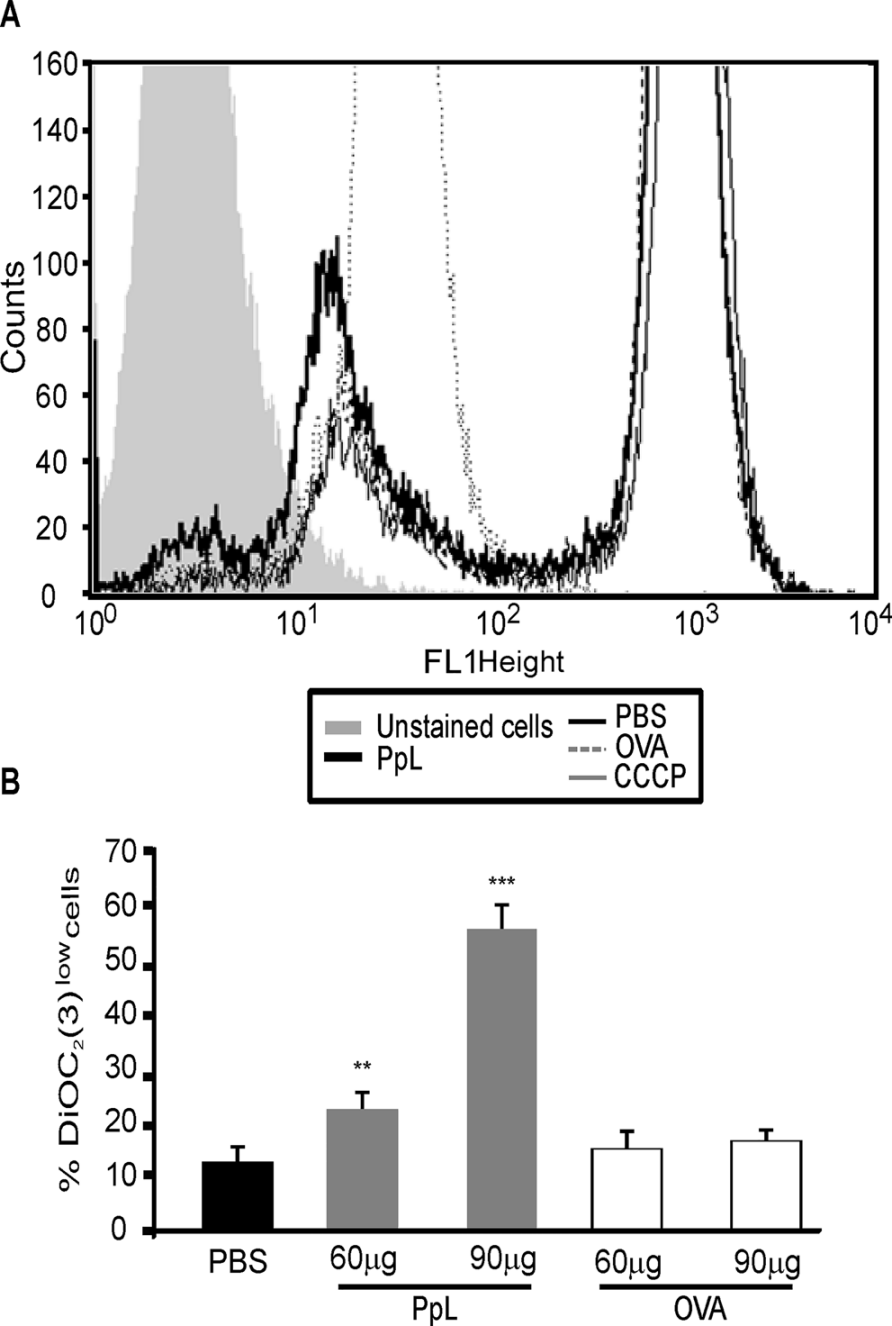
PpL induces increases in the percentage of DiOC_2_(3)^low^ Daudi cells. Cells were incubated with different doses of PpL, OVA or PBS. After 72 hs, the ΔΨm was measured by FACS using DiOC_2_(3) staining. **A)** Representative overlaid histograms for DiOC_2_(3) fluorescence using 90 μg of PpL. **B)** Percentage of DiOC_2_(3)^low^ cells (mean ± SEM) of three independent experiments, (** p<0.01 PpL (60μg/ml) vs PBS; *** p<0.001 PpL (90 μg/ml) vs PBS).

We investigated whether PpL is able to induce apoptosis of Daudi cells. As can be observed in Fig 5A and 5B, PpL induced increases in the percentage of Daudi cells with low DNA content. The levels of DNA content were measured in the presence of caspase inhibitors. As shown in Fig 5C, whereas caspase-9 and 3 inhibitors significantly decreased the percentage of Daudi cells with low DNA content, no alterations were observed in the presence of caspase-8 inhibitor. PpL also induced an increase in the percentage of Annexin V+ Daudi cells. No alterations in the percentage of apoptosis could be detected in the presence of caspase-8 inhibitor whereas caspase-9 inhibitor abrogated PpL-induced apoptosis (Fig 6). These results led us to determine the effect of PpL in the mitochondrial membrane potential. As can be observed in Fig 7, a significant increase in the percentage of DiOC_2_(3)^low^ Daudi cells was observed when cells were incubated with different doses of PpL, indicating that this Sag is able to alter the ΔΨm in Daudi cells. Together, these data suggest that the intrinsic pathway is involved in PpL-induced apoptosis in Daudi cells.

**Fig 8:**
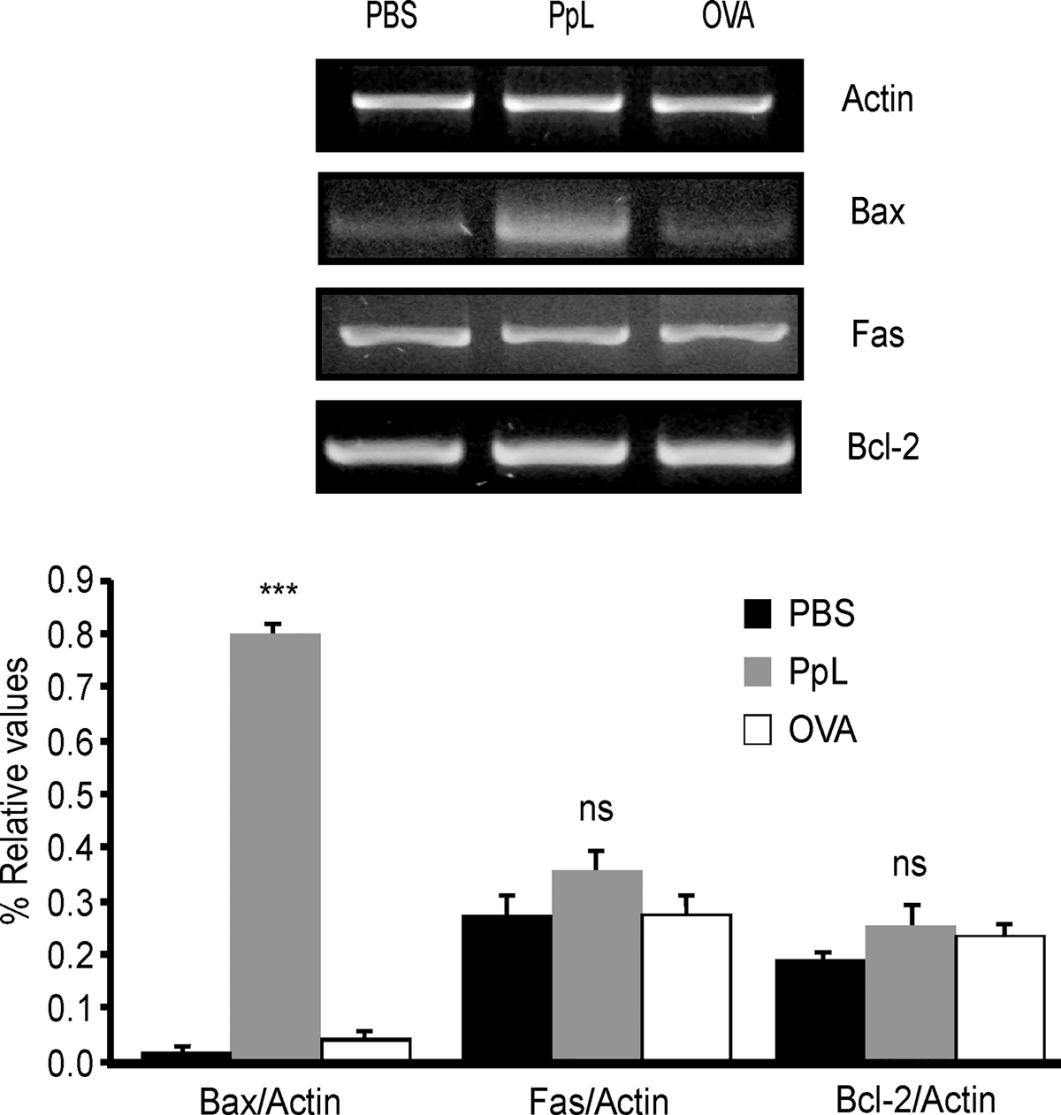
PpL induces increases in Bax but not in Bcl-2 or Fas mRNA. Daudi cells were treated with PpL (100 μg/ml), OVA (100 μg/ml) or PBS. RNA was extracted and cDNA was obtained by RT-PCR and analyzed by semi-quantitative PCR for Bax, Fas, Bcl-2 and actin. Image of a representative result is shown. The OD of the bands of Bax, Bcl-2 and Fas cDNA was quantified and normalized to actin. The relative levels are shown. Data are presented as the mean ± SEM of four independent experiments, (*** p<0.001 PpL vs PBS; ns p>0.05 PpL vs PBS).

**Fig 9:**
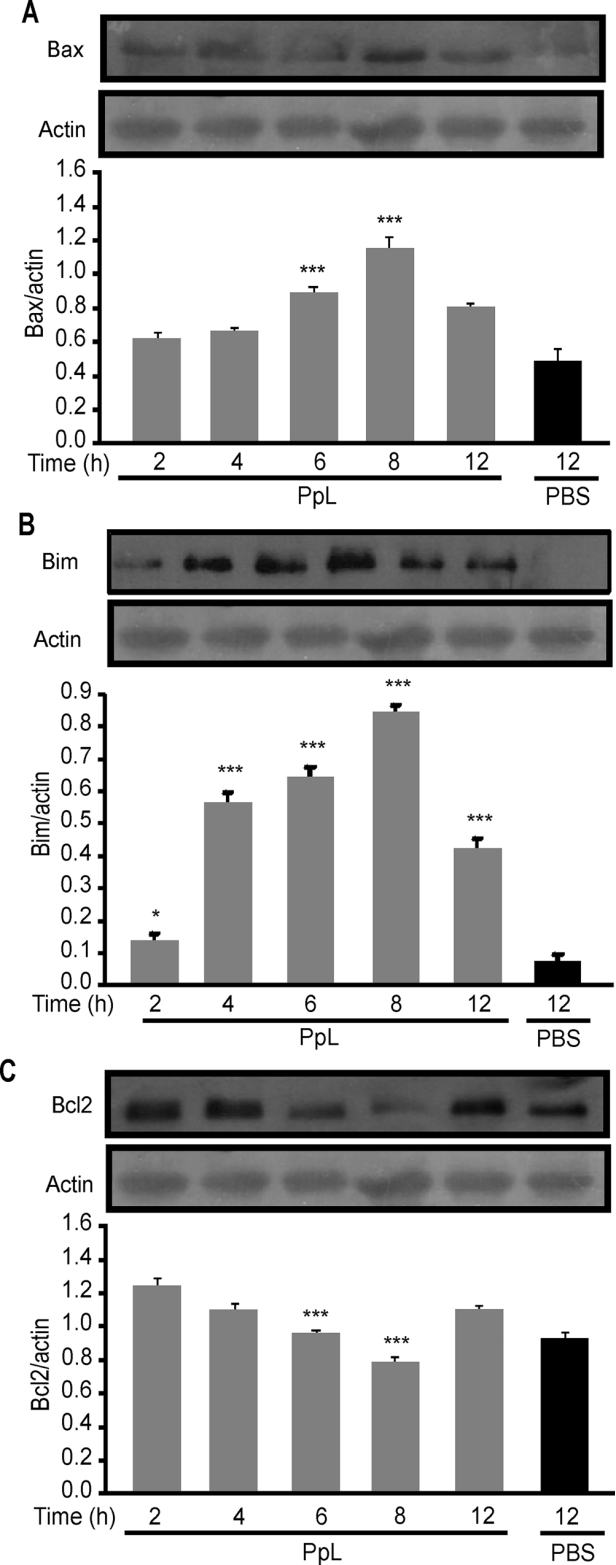
PpL induces increases of Bim and Bax and decreases of Bcl-2 proteins. Daudi cells were incubated in the presence of PpL (100 μg/ml) or PBS for the indicated times. Total proteins were obtained and Western blots were performed. Membranes were sequentially blotted with anti-Bax **(A),** anti-Bim **(B)**, anti-Bcl-2 **(C)**, or anti-actin antibodies. Images of representative Western blots are shown. The optical density of Bax, Bim and Bcl-2 bands were quantified and normalized to actin. The relative levels are shown. Data are presented as the mean ± SEM of three independent experiments (* p<0.05 *** p<0.001).

**Fig 10:**
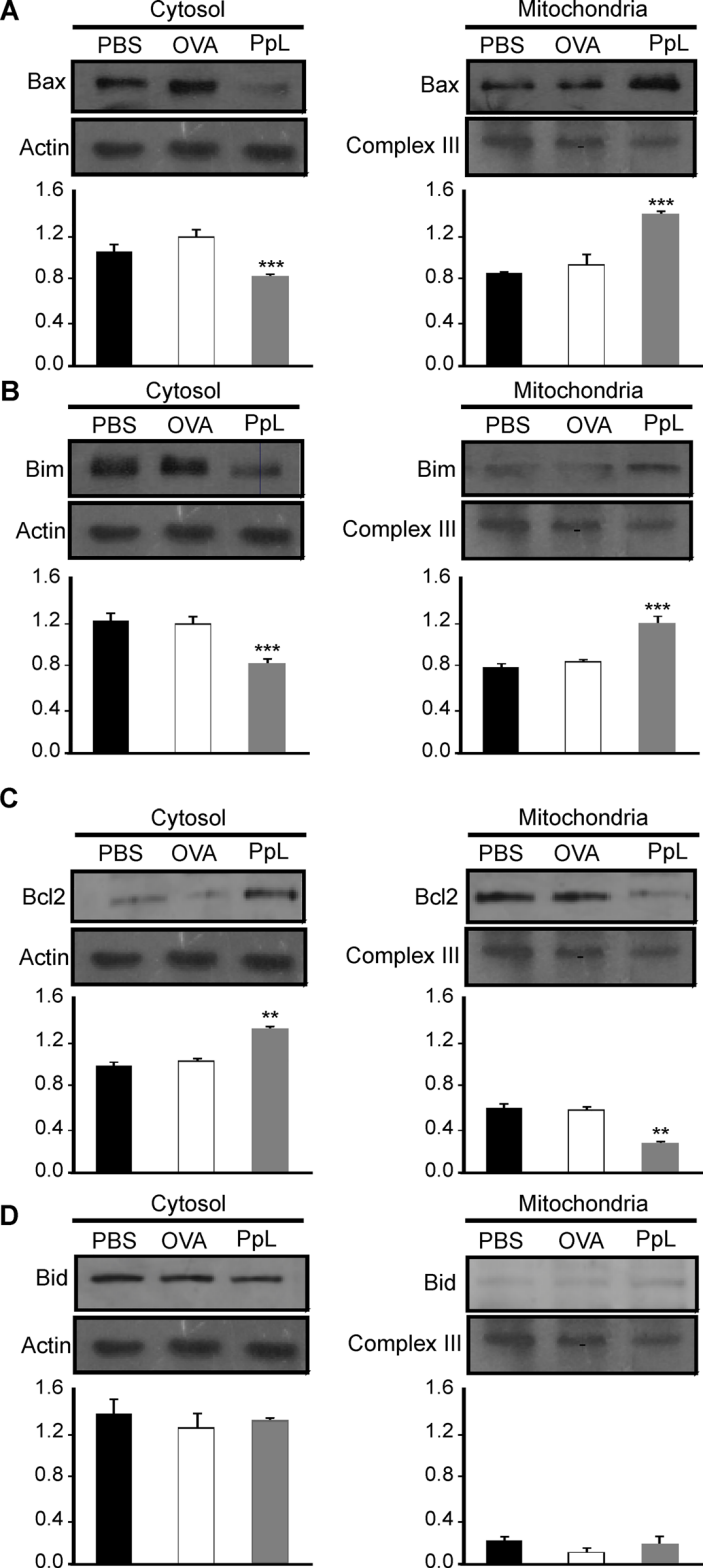
Translocation of Bim and Bax to the mitochondria and of Bcl2 to the cytosol. Daudi cells were incubated in the presence of PpL (100 μg/ml) or PBS for 8 hs. Cell lysates containing cytosol and mitochondria were prepared. Expression of Bax, Bim, Bcl2 and Bid were analyzed by Western blotting. Membranes were sequentially blotted with anti-Bax **(A),** anti-Bim **(B)**, anti-Bcl-2 **(C)**, anti-Bid **(D)**, anti-actin or anti-III complex antibodies. Images of representative Western blots are shown. The optical density of Bax, Bim, Bcl-2 and Bid bands were quantified and normalized to actin or anti-III complex. The relative levels are shown. Data are presented as the mean ± SEM of three independent experiments (** p<0.01; *** p<0.001).

**Fig 11:**
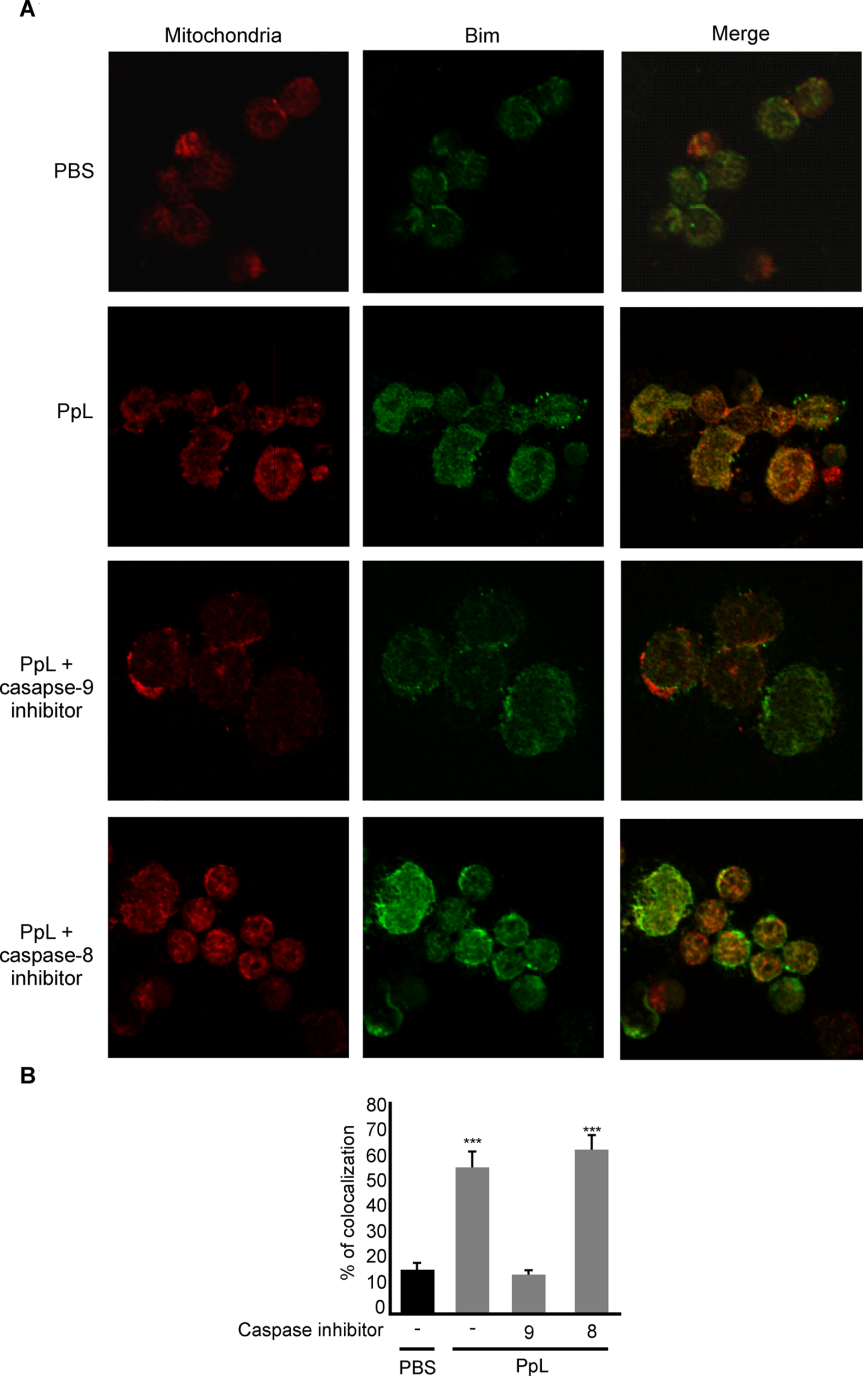
PpL induces Bim colocalization with mitochondria. After 8 hs of pre-incubation with or without inhibitors of caspase-9 or -8, Daudi cells were treated with PpL (100 μg) or PBS for 8 hs. **(A)** Representative immunofluorescences showing colocalization between mitochondria (Red) and Bim protein (green) (See Materials and Methods). Signal overlap was quantified using MBF-Image J **(B)**. Pearsons colocalization coefficients were calculated from three independent experiments and then converted to percentages, (*** p<0.001).

**Fig 12:**
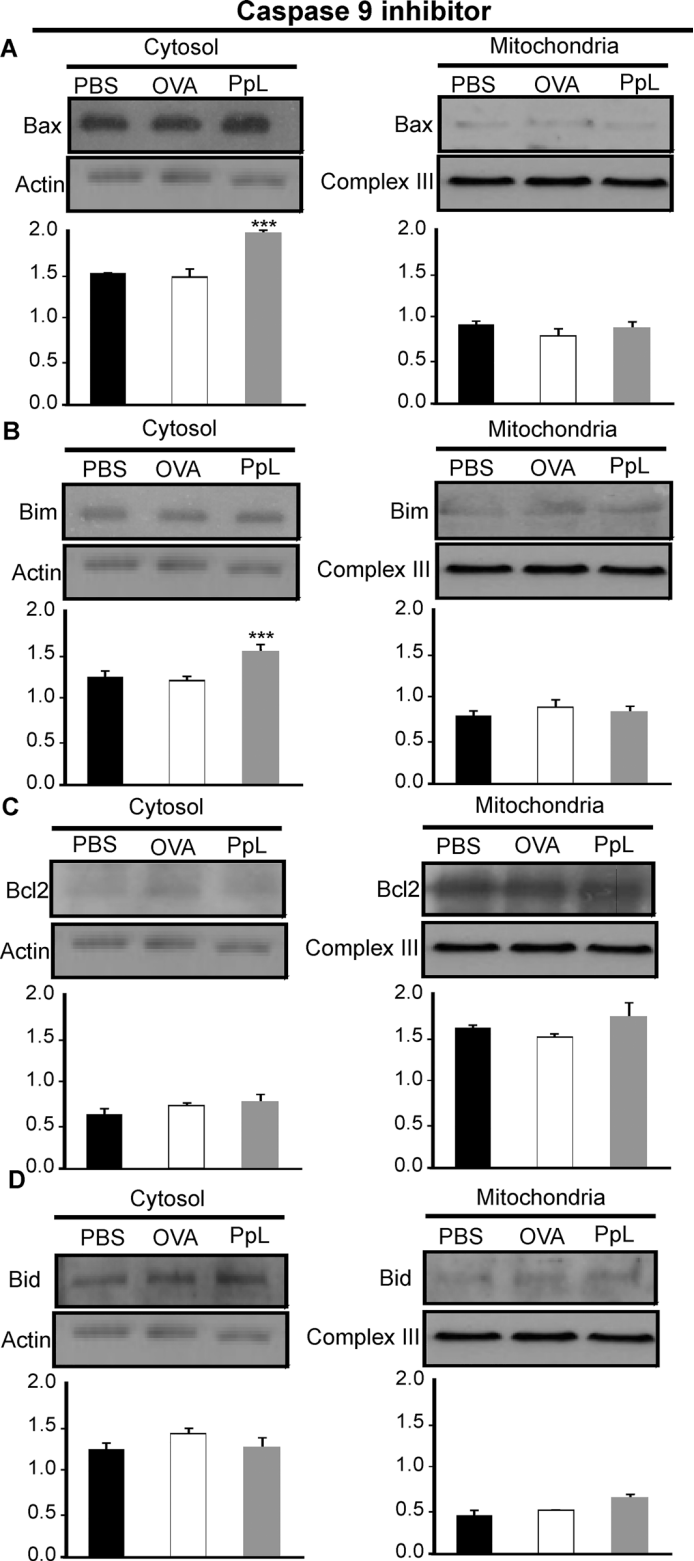
Effect of caspase-9 inhibitor on the PpL-induced translocation of Bax, Bim and Bcl-2 between the mitochondrial and cytosol compartments. Daudi cells pretreated with caspase-9 inhibitor were incubated with PpL (100 μg/ml), OVA (100 μg/ml) or PBS for 8 hs. Cell lysates containing cytosol and mitochondria fractions were prepared. Expression of Bax, Bim, Bcl-2 and Bid was analyzed by Western blotting. Membranes were sequentially blotted with anti-Bax **(A)**, anti-Bim **(B),** anti-Bcl-2 **(C)**, anti-Bid **(D)**, anti-actin or anti-III complex antibodies. Images of representative Western blots are shown. The optical density of Bax, Bim, Bcl-2 and Bid bands was quantified and normalized to actin or III complex. The relative levels are shown. Data are presented as the mean ± SEM of three independent experiments, (*** p<0.001).

**Fig 13:**
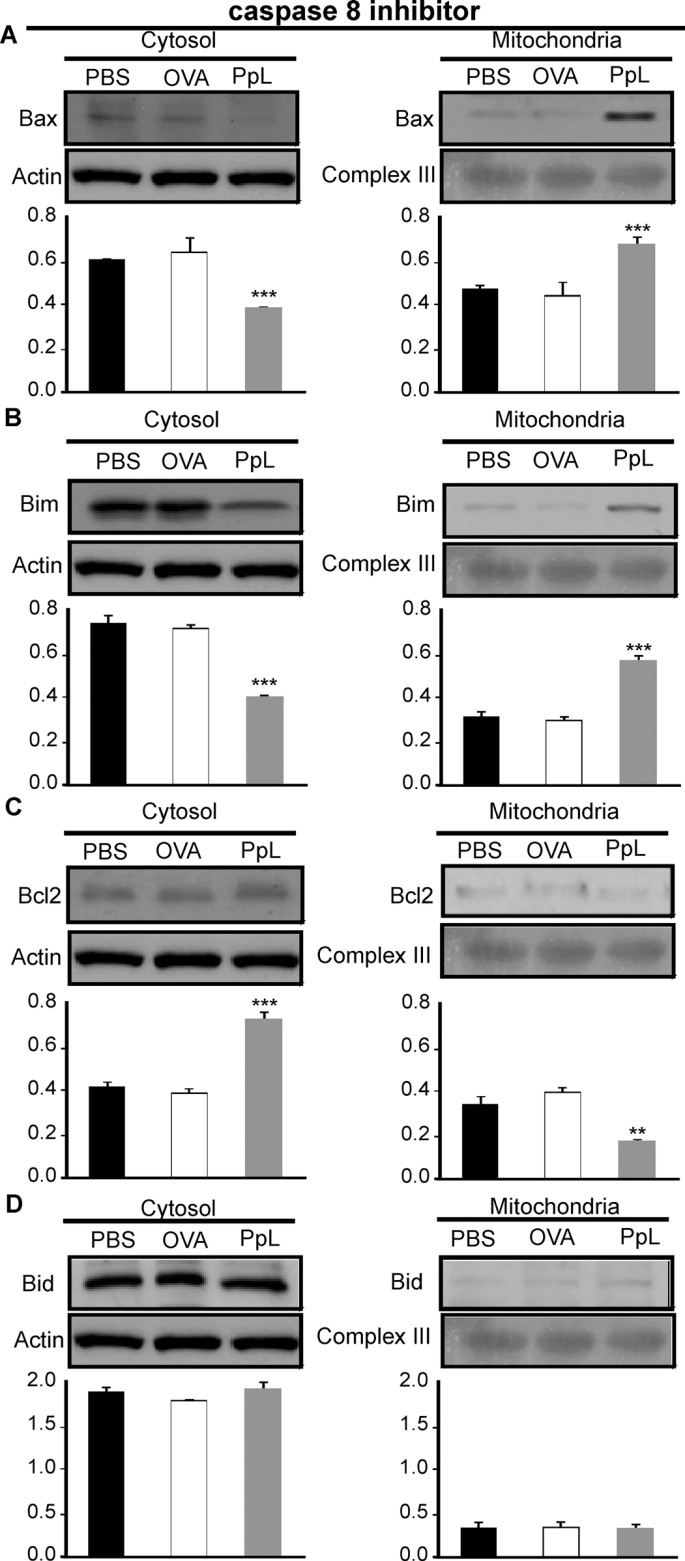
Effect of caspase-8 inhibitor on the PpL-induced translocation of Bax, Bim and Bcl-2 between the mitochondrial and cytosol compartments. Daudi cells pretreated with caspase-8 inhibitor were incubated with PpL (100 μg/ml), OVA (100 μg/ml) or PBS for 8 hs. Cell lysates containing cytosol and mitochondria fractions were prepared. Expression of Bax, Bim, Bcl-2 and Bid was analyzed by Western blotting. Membranes were sequentially blotted with anti-Bax **(A)**, anti-Bim **(B)**, anti-Bcl-2 **(C)**, anti-Bid **(D)**, anti-actin or anti III complex antibodies. Images of representative Western blots are shown. The optical density of Bax, Bim, Bcl-2 and Bid bands was quantified and normalized to actin or III complex. The relative levels are shown. Data are presented as the mean ± SEM of three independent experiments, (** p<0.01 PpL vs PBS; *** p<0.001 PpL vs PBS).

It has been shown that the B-cell Sag SpA causes activation-induced apoptosis *in vivo* by a process that requires the induction of the pro-apoptotic Bim member of the Bcl-2 family [24]. We examined whether pro- and anti-apoptotic proteins involved in the intrinsic pathway of apoptosis were altered by PpL. As shown in Fig 8, an important increase of Bax mRNA was found, whereas no differences in the level of Bcl-2 mRNA could be recorded. Protein expression of Bax, Bim and Bcl-2 were then measured in whole homogenates from saline- and PpL-incubated Daudi cells. Exposition to PpL induced significant increases in the levels of Bax and Bim proteins and a significant decrease in the level of Bcl-2 (Fig 9). In addition, the mitochondrial and cytosolic fractions were isolated in order to measure Bax and Bim translocation to the mitochondria and Bcl-2 translocation to the cytosol. As can be seen in Fig 10, the levels of mitochondrial Bim and Bax proteins were significantly increased by PpL exposition, whereas a decrease in the level of these proteins was observed in the cytoplasmic fraction. Increases in mitochondrial Bim were also detected using confocal immunofluorescence (Fig 11). Regarding the anti-apoptotic protein Bcl-2, a significant increase of this protein in the cytoplasmic fraction and a decrease in the mitochondrial fraction were detected (Fig 10). Bim and Bax translocation to the mitochondria decreased in the presence of caspase-9 inhibitor (Figs 11 and 12) but not in the presence of caspase-8 inhibitor (Figs 11 and 13). As expected, a decrease in Bcl-2 translocation to the cytosol was observed in the presence of caspase-9 inhibitor (Fig 12) but not in the presence of caspase-8 inhibitor (Fig 13). These data indicate that the intrinsic pathway is involved in PpL-induced apoptosis in Daudi cells.

No alterations in the levels of Fas mRNA were found (Fig 8). No alterations in the levels of Fas and Fas-L were observed using flow cytometry (data not shown). No differences in the level of expression of Bid protein could be detected (Figs 10, 12 and 13). Besides, truncated Bid (tBid) could not be detected. These results and the lack of effect of caspase 8-inhibitor in the level of apoptosis suggest that PpL does not activate the extrinsic pathway of apoptosis.

Overall, our data suggest that the intrinsic but not the extrinsic pathway is involved in PpL- induction of apoptosis in malignant B cells.

## Discussion

B-cell Sags bind to the Fab regions of Ig molecules outside their complementarity-determining regions [3, 4]. These unconventional antigens interact with many members of an entire V_H_ or V_L_ gene family [4, 25]. Although the ability of B-cell Sags to induce apoptosis of normal B-cells has been described in normal murine and human B lymphocytes [6, 24, 26, 27], no attempts to study their effects on malignant B cells have been reported.

In the present study, we have investigated whether PpL -a B-cell Sag interacting with normal lymphocytes expressing the κ light chain in their BCR- is able to induce the apoptosis of κ+ malignant B cells using spontaneous murine lymphoma B cells and Daudi cells.

PpL was found to induce an early decrease in the MFI of the κ+ chain both in murine and human malignant B cells *in vitro*. PpL was also able to increase the levels of expression of co-stimulatory molecules such as CD80 and CD86 only in κ+ positive malignant B cells. These results suggest that the interaction between PpL and κ+ B-cell lymphomas activates cognate malignant B cells as it has been reported for normal κ+ cells [23, 28]. Of importance, PpL did not increase the proliferative levels of malignant B cells.

Apoptosis is a highly regulated form of cell death that controls normal homeostasis. The inactivation of apoptosis is central to the development of cancer. This disabling of apoptotic responses might be a major contributor to treatment resistance [29]. In this work, we show that PpL is able to decrease the DNA content both in murine and human lymphoma κ+ cells *in vitro*, suggesting that it causes the apoptosis of these cells. Noalteration in the DNA content was registered in a λ+ murine B-cell lymphoma. Moreover, an *in vivo* experiment using murine malignant κ+ B cells confirmed that PpL induces the apoptosis of these cells. Using Annexin V we also showed that PpL was able to induce the apoptosis of Daudi cells. Our results show that, as reported for normal κ+ B cells [30], PpL is able to alter the ΔΨm both in murine and human lymphoma cells, suggesting that the intrinsic pathway is involved in PpL-induced apoptosis. Induction of apoptosis in Daudi cells was not altered in the presence of a caspase-8 inhibitor. No alterations in the levels of expression of Fas and Fas-L were found. The level of Bid was not altered and tBid was not detected. These data suggest that PpL does not activate the extrinsic pathway of apoptosis.

The mitochondrial apoptotic pathway is controlled by the pro- and anti-apoptotic proteins of the Bcl-2 family [31]. The members of this family can be divided into three classes based on their sequence homology and function [32]. The first class comprises the anti-apoptotic proteins Bcl-2, Bcl-w, Mcl-1, Bfl-1, and Bcl-xl which contain all four Bcl-2 homology domains (BH1–4). The proteins in this class bind and sequester their pro-apoptotic counterparts thus preventing apoptosis. The second class includes the pro-apoptotic proteins Puma, Bim, Bid, Bad, Bik, Noxa, and Bmf, which contain only the BH3 domain (BH3-only) [33]. The final class contains Bcl-2-associated x protein (Bax) and Bcl-2 antagonist or killer (Bak) which contains BH1–3 domains [34]. Bax and Bak, when activated, oligomerize and cause mitochondrial outer membrane permeabilization. It has been reported that Bid preferentially activates Bak while Bim preferentially activates Bax, affecting chemotherapy response [35]. Other authors showed that most BH3-only proteins can directly activate Bak and Bax, and show no preference for Bak versus Bax [36].

The participation of the intrinsic or mitochondrial pathway in PpL- induced apoptosis of Daudi cells was indicated by: i) decreases of apoptosis in the presence of caspase-9 inhibitor; ii) significant increases of Bim and Bax proteins and downregulation of Bcl-2, thereby increasing the Bax/Bcl-2 expression ratio; iii) the translocation from the cytoplasm to the mitochondria of Bax and Bim pro-apoptotic proteins and its inhibition by caspase-9 inhibitor and iv) translocation of Bcl-2 protein from the mitochondria to the cytosol and its inhibition by caspase-9 inhibitor.

A significant decrease of Bcl-2 protein levels along with no alterations in their mRNA level raises the possibility that alterations induced by PpL would include increases in Bcl-2 degradation [37].

Overall, our data demonstrate that the mitochondrial pathway is involved in Sag-induced apoptosis in B-cell malignancies.

Previously, we had shown that T-cell Sags are able to induce the apoptosis of cognate murine T-cell lymphomas both *in vitro* and *in vivo*, being the apoptosis pathways involved, the same as those described for normal cognate T cells [14].

Results reported herein show that PpL induces the apoptosis of malignant murine and human k+ B-cell lymphomas both *in vitro* and *in vivo* using the intrinsic apoptotic pathway as suggested for normal B lymphocytes.

It has been suggested that B-cell Sags could be involved in the development and evolution of some CLL clones [38, 39]. On the other hand, Silverman and Goodyear have hypothesized that B-cell Sags might provide a new therapeutic approach for the treatment of B-cell neoplastic diseases [40].

Further studies are needed to clarify the *in vivo* relevance of the present findings. However, our results suggest that B-cell Sags could be envisaged as a therapeutic tool for B-cell malignancies. The use of Sags as therapeutic agents in B-cell neoplastic diseases expressing functional B-cell receptors would have the advantage of deleting restricted B-cell clones without causing the death of other normal cells.

## Supporting Information

S1 Fig PpL decreases the expression of the κ chain and induces the apoptosis of normal κ+ B cells. Splenocytes from normal BALB/c mice were incubated with PpL (100 μg/ml), OVA (100 μg/ml) or PBS for 1 hr. **A)** Representative overlaid histograms of κ chain expression. **B)** Representative contour plots of Annexin V staining and **C)** Percentage of apoptotic cells (mean ± SD, n = 3) (*** p<0.001 PpL vs PBS).
(TIF)

S2 Fig PpL does not induce the expression of κ chain in LBO cells. LBO cells were incubated with PpL (100 μg/ml), OVA (100 μg/ml) or PBS for 1 hr. Representative overlaid histograms of κ chain expression are shown. Experiments were performed three times with similar results, (*** p<0.001 PpL vs PBS).
(TIF)
