# Supplementary figures and images for: A B-Cell Superantigen Induces the Apoptosis of Murine and Human Malignant B Cells
Source: PLoS One. 2016 Sep 7;11(9):e0162456. doi: 10.1371/journal.pone.0162456 (PMC5014328; doi:10.1371/journal.pone.0162456)

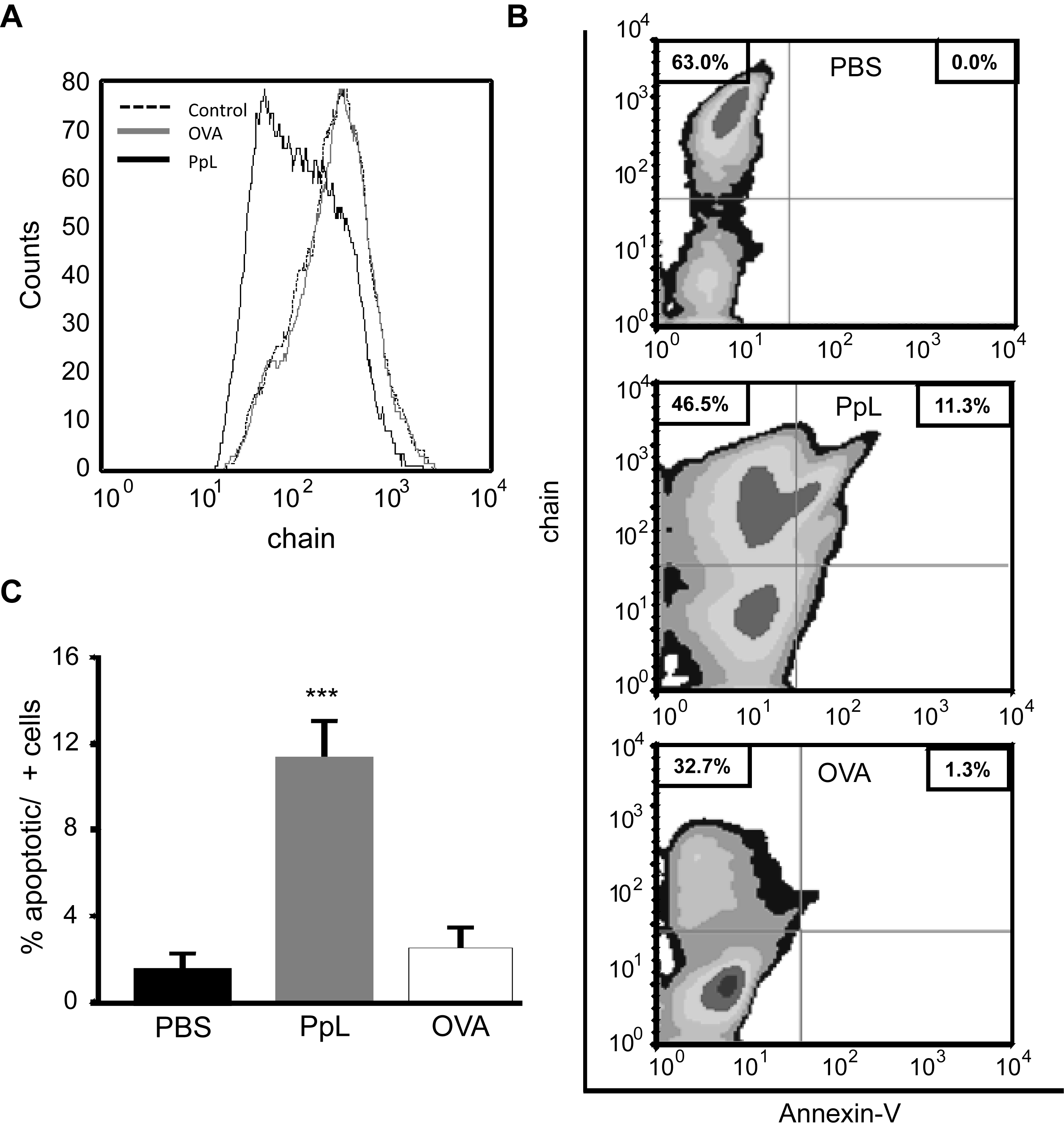

Supplement: S1 Fig — Splenocytes from normal BALB/c mice were incubated with PpL (100 μg/ml), OVA (100 μg/ml) or PBS for 1 hr. A) Representative overlaid histograms of κ chain expression. B) Representative contour plots of Annexin V staining and C) Percentage of apoptotic cells (mean ± SD, n = 3) (*** p<0.001 PpL vs PBS). (TIF) [file pone.0162456.s001.tif]

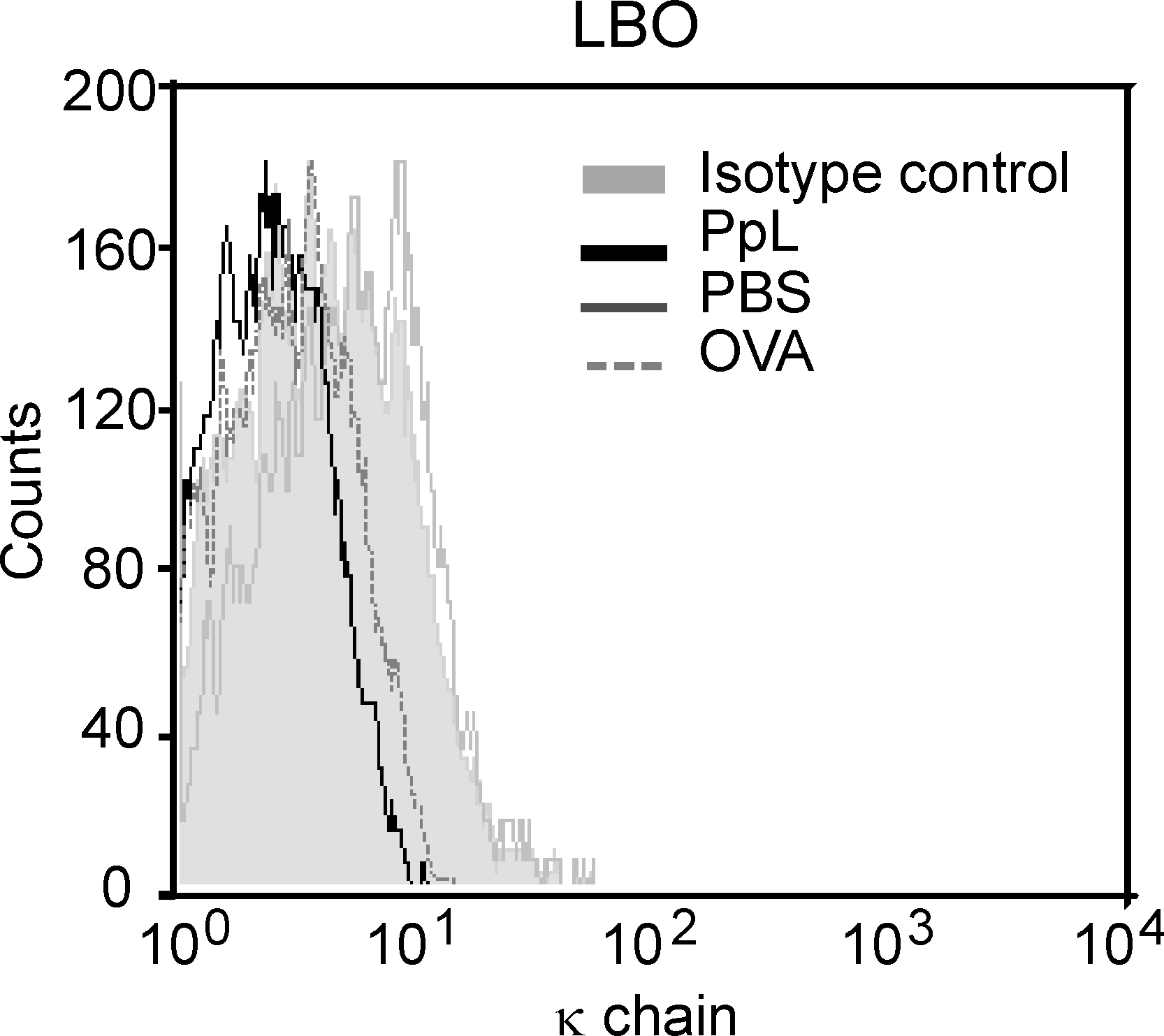

Supplement: S2 Fig — LBO cells were incubated with PpL (100 μg/ml), OVA (100 μg/ml) or PBS for 1 hr. Representative overlaid histograms of κ chain expression are shown. Experiments were performed three times with similar results, (*** p<0.001 PpL vs PBS). (TIF) [file pone.0162456.s002.tif]
